# Supplementary material for: Chromothripsis during telomere crisis is independent of NHEJ, and consistent with a replicative origin
Source: Genome Res. 2019 May;29(5):737–49. doi: 10.1101/gr.240705.118 (PMC6499312; doi:10.1101/gr.240705.118)
Supplement: Supplemental Material [file supp_gr.240705.118_Supplemental_file_1.zip › contigs/annotated_contigs/DB55/contig.4.DB55_length_298_mean_cov_4.53020134228.docx]

**DB55_length_298_mean_cov_4.53020134228**

```
TCTGTTGTCTTTAATAAAGAGCAGTGTCTTCCTCCCTCAGTGCCCATCTCTTTGCACCTGCCACTCTGGCTGCTGCTCACACCACTGCA
 >chrUn_gl000212:112572-112797 - E=1e-120
CAGAAACCGCTTTCTCCAGACTTACCGGCAACCACCTCAGCAGCACAGCTGTTGCTTCTCAAACTTTAATGTGCATCAGAATCAATTGG

AGGGCTCATGAGCCTGCAGGTTGCTGAGCCTTACTCACAGTTTTTCT|TGTGCCTGACCTGGAAGTGGATTTTGAAGTGGATTTTGCAG
                                                 >chr19:19483610-19483683 + E=3e-32
CCTGTTTGTGTCTCCCAAGCCACAAAAGACTC|GAAATAGATTATTACCTACGGATT|TTTTCTGTCTTCATGACATATATAATTTTTT
                                                         >chr7:53883892-53884121 - E=4e
CTTAAATTTTTATTATATTTCTAAGCTATGCTATTTGCAAGTTTTTTTTCAAATTGTCACATATTCCCTAAAAAAGTATTTTAATATAT
-123
TTGTTGAAAAGAAATTTGTGTGTATGTGATCCCATGCAGTTTAAACTGGTGTTGTTCAAGGGTCCACCGTATATCGTGGTCAGAATTCC

TCCTCAGGGTGATTGGATT|C|CCGGCTAATTTTTGTATTTTTGGTAGGGACGGGGTTTCACCATGT                    >chr7:53869119-53869165 - E=5e-16
```
